# Case Report: Successful treatment of recurrent COVID-19 with intravenous immunoglobulin in a patient with rituximab-induced B-cell depletion and restoration of Fc-mediated effector functions

**DOI:** 10.3389/fimmu.2026.1797070

**Published:** 2026-05-25

**Authors:** Hee Bum Jo, Jong Su Kang, Sungim Choi, Seong Yeon Park

**Affiliations:** 1 Division of Infectious Diseases, Department of Internal Medicine, Incheon Sejong Hospital, Incheon, Republic of Korea; 2 Division of Infectious Diseases, Department of Internal Medicine, Dongguk University Ilsan Hospital, Goyang-si, Gyeonggi-do, Republic of Korea

**Keywords:** B-cell depletion, COVID-19, Fc effector function, intravenous immunoglobulin, rituximab

## Abstract

We report the case of a 47-year-old woman with neuromyelitis optica spectrum disorder who developed prolonged coronavirus disease 2019 (COVID-19) pneumonia 3 months after receiving rituximab. Rituximab-induced B-cell depletion left her highly susceptible to persistent viral infection, resulting in three serial hospital admissions due to recurrent clinical deterioration despite standard antiviral therapy. During her third admission, her clinical status and radiographic findings continued to decline even after remdesivir and dexamethasone were reinitiated. As salvage therapy, she received a 3-day course of intravenous immunoglobulin (IVIG), resulting in rapid improvement and complete resolution of oxygen requirement. To investigate the therapeutic mechanism, we conducted longitudinal serologic analyses. Before IVIG administration, severe acute respiratory syndrome coronavirus 2 (SARS-CoV-2)–specific IgG was undetectable, and antibody-dependent cellular cytotoxicity (ADCC) and antibody-dependent cellular phagocytosis (ADCP) activities were minimal. Following IVIG infusion, IgG titers against both wild-type and Omicron variants increased substantially. Notably, Fc-mediated effector functions, including ADCC and ADCP, were restored and peaked 1 week after treatment, aligning with the patient’s clinical recovery. These findings support the mechanistic hypothesis that IVIG benefits individuals with B-cell depletion not only through passive antibody transfer but also by contributing to the restoration of critical immune effector functions. This case suggests that IVIG could be considered as a potential therapeutic adjunct for managing persistent COVID-19 in patients following B-cell–depleting therapy.

## 1 Introduction

Since its emergence in late 2019, coronavirus disease 2019 (COVID-19) has posed an unprecedented global health challenge, with clinical manifestations ranging from asymptomatic infection to severe pneumonia and acute respiratory distress syndrome (1). Current therapeutic strategies include antivirals such as remdesivir, dexamethasone, interleukin-6 inhibitors, and Janus kinase inhibitors for severe disease (2). However, the clinical course and recovery are strongly influenced by host immune competence, particularly the integrity of humoral immunity, which plays a crucial role in viral clearance and the prevention of disease progression (3, 4).

Patients receiving B-cell–depletion therapy, particularly rituximab—an anti-CD20 monoclonal antibody widely used to treat hematologic malignancies and autoimmune disorders—face unique challenges when infected with SARS-CoV-2 (5–7). Rituximab induces marked and prolonged B-cell depletion, resulting in impaired antibody production that can persist for months to years after treatment cessation (8). This immunocompromised state increases susceptibility to persistent or relapsing COVID-19 due to inadequate viral clearance, even after resolution of initial symptoms (5, 6). Standard antiviral therapies, including remdesivir, have shown limited efficacy as monotherapy in this population, and evidence-based management strategies for individuals experiencing B-cell depletion with protracted infection are still lacking (7).

Notably, the continuous evolution of SARS-CoV-2 has led to the successive emergence of variants, such as the Omicron sublineages, which exhibit broad resistance to most previously authorized monoclonal antibody therapeutics (9). This evolving viral landscape poses a persistent challenge for immunocompromised patients. These individuals often lack endogenous antibodies and can no longer rely on monoclonal therapies or vaccines to achieve viral clearance. Consequently, there is a critical need to evaluate alternative passive immunotherapy approaches, such as pooled intravenous immunoglobulin (IVIG), which contains a diverse repertoire of neutralizing antibodies and may restore essential immune effector functions (10–13).

In this report, we evaluate the clinical and immunological impact of IVIG in a patient with rituximab-induced B-cell depletion experiencing persistent COVID-19. To elucidate the potential mechanisms underlying recovery in the absence of humoral immunity, we assessed the longitudinal restoration of Fc-mediated effector functions, including antibody-dependent cellular cytotoxicity (ADCC) and antibody-dependent cellular phagocytosis (ADCP), alongside SARS-CoV-2–specific antibody titers before and after IVIG administration.

## 2 Case description

### 2.1 First and second admission (August 21, 2023 and August 30, 2023)

A 47-year-old woman had been diagnosed with neuromyelitis optica spectrum disorder 8 years prior. She was being maintained on rituximab therapy (1,000 mg every 6 months) to prevent relapses, with her most recent dose administered three months before the current episode. At baseline, she was fully ambulatory without assistance, with an Expanded Disability Status Scale score of 2.0, reflecting only mild residual visual impairment. She had no other comorbidities, such as obesity (Body Mass Index 21.23 kg/m²), diabetes, or hypertension, and there was no documented history of hypogammaglobulinemia prior to the COVID-19 infection.

In late July 2023 (Day 0), she developed COVID-19 symptoms following family exposure, marking the beginning of a 70-day clinical course that aligns with the definition of persistent SARS-CoV-2 infection (7). This case followed a distinct remitting-relapsing pattern, where periods of clinical stability were followed by recurring pneumonia due to incomplete viral clearance in a B-cell-depleted host. The illness followed a distinct remitting-relapsing pattern; after an initial period of clinical stability, she developed a recurring cough and fever in mid-August. On Day 22 (August 21), she was diagnosed with COVID-19 via a rapid antigen test and admitted for her first hospitalization due to pneumonia (Hospitalization 1).

**Figure 1:**
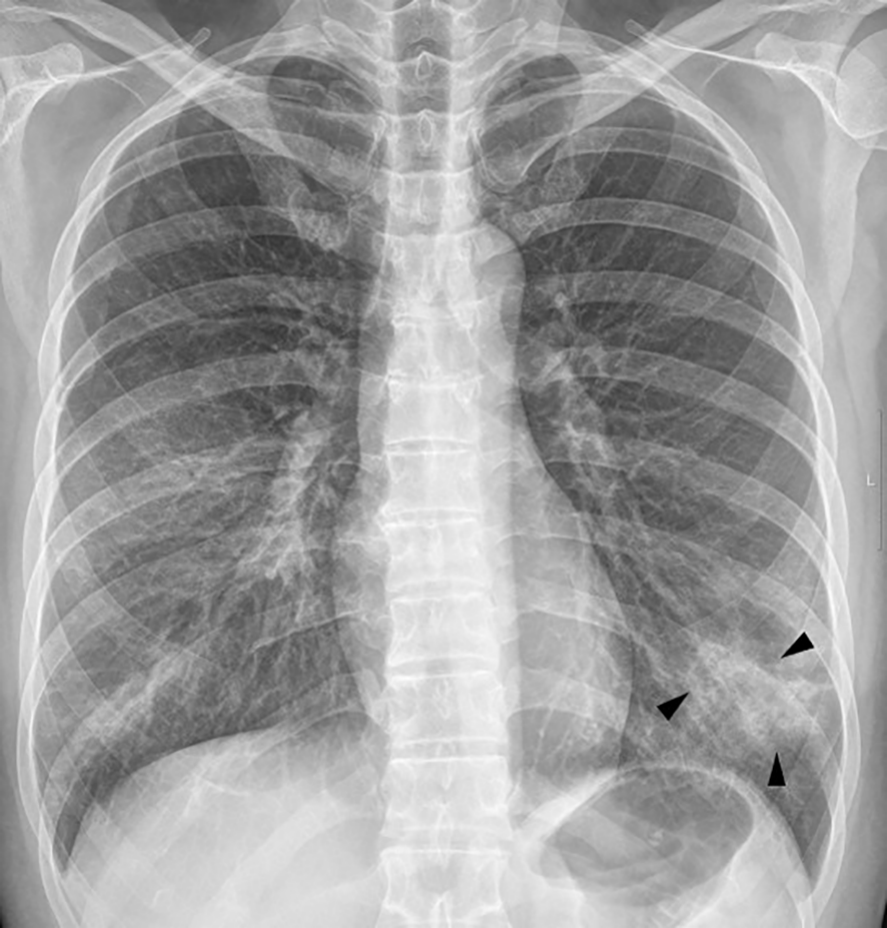
Chest radiograph at initial admission. Posteroanterior chest radiograph obtained on hospital day 1 shows consolidation in the left lower lung zone. A subtle ground-glass opacity (GGO) is also visible in the right middle–lower lung zone.

**Figure 2:**
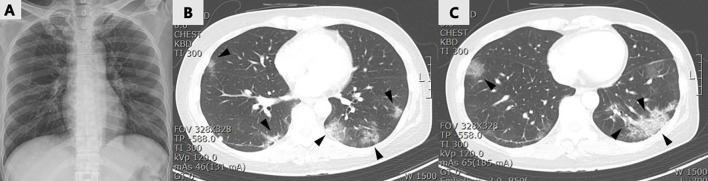
Radiologic findings during the second admission. **(A)** Chest radiograph showing no definitive active lung lesion. **(B, C)** Axial chest computed tomography (CT) images showing bilateral patchy consolidations and GGOs with associated interstitial thickening.

Upon admission, initial laboratory findings revealed a white blood cell (WBC) count of 5,760/mm^3^ (lymphocytes 9.9%, absolute lymphocyte count 570/mm^3^), and elevated inflammatory markers, including C-reactive protein (8.16 mg/dL), lactate dehydrogenase (381 U/L), and ferritin (585 ng/mL). Chest radiography confirmed the presence of pneumonia (**Figure 1**). She received a 5-day course of remdesivir and dexamethasone (6 mg daily), which resulted in symptomatic relief and radiographic improvement. The patient was subsequently discharged on hospital day 8. However, this recovery was transient; only two days after discharge, she was readmitted with recurrent fever (38.7 °C) tachycardia (131 beats/min), and worsening dyspnea (Day 31, Hospitalization 2). Chest computed tomography (CT) revealed worsening bilateral patchy consolidations and ground-glass opacities (GGOs), indicative of worsening COVID-19 pneumonia (**Figures 2A–C**). Laboratory tests at this time confirmed persistent lymphopenia (8.1%) and a further rise in ferritin (781 ng/dL), despite a relatively lower CRP (3.16 mg/dL). Remdesivir and dexamethasone were re-initiated, resulting in temporary clinical improvement, and she was discharged on day 10 with a tapered dose of dexamethasone.

### 2.2 Third admission (September 16, 2023)

**Figure 3:**
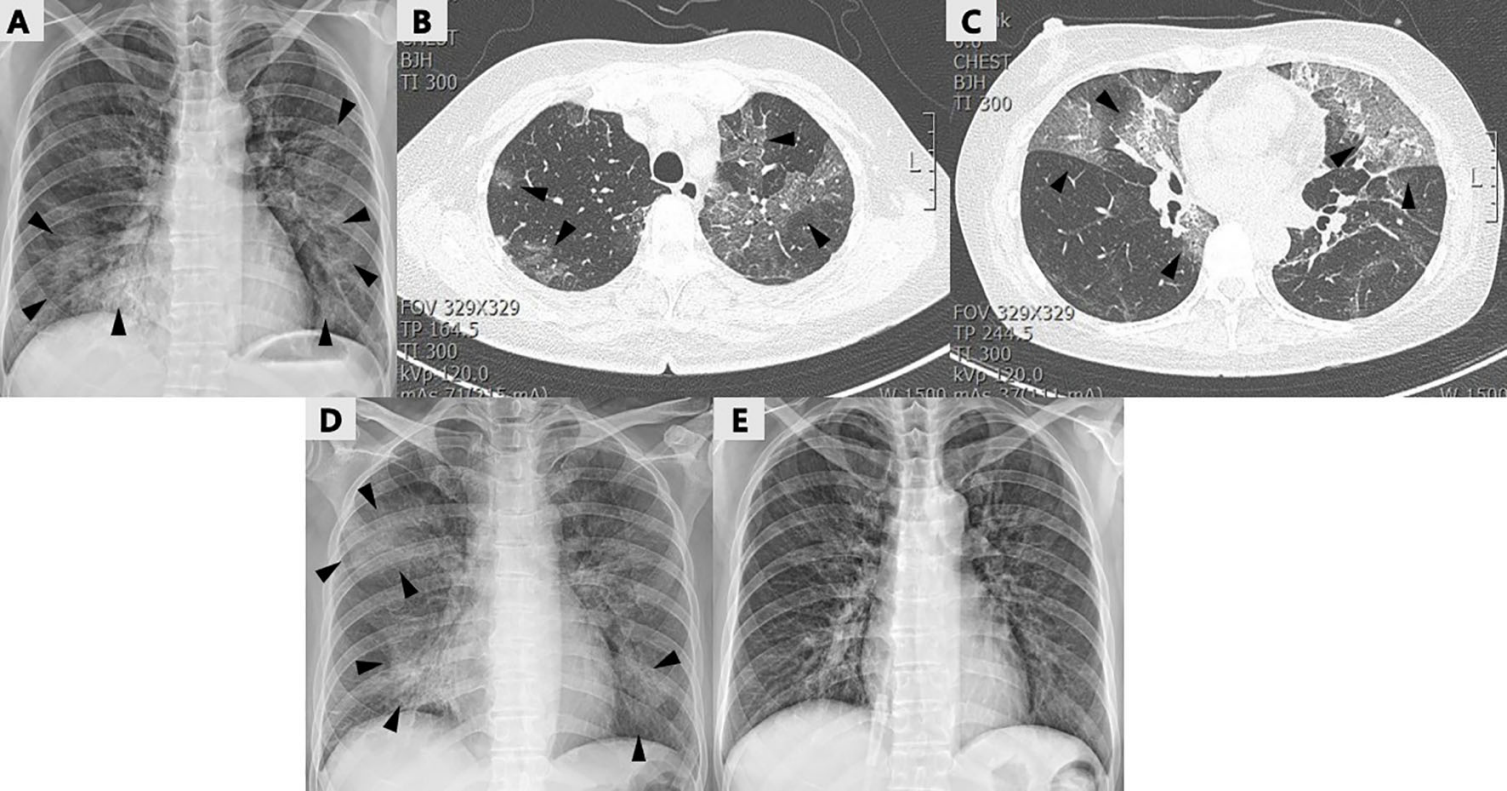
Radiologic progression and clinical response to intravenous immunoglobulin (IVIG). **(A)** Chest radiograph showing multifocal consolidations in both lungs. **(B, C)** Axial chest CT images showing bilateral patchy consolidations and GGOs with interstitial thickening. **(D)** Follow-up chest radiograph showing worsening multifocal consolidations and GGOs, accompanied by fever during hospitalization, prompting initiation of IVIG. **(E)** Chest radiograph at discharge showing significant resolution of lung lesions after IVIG treatment.

**Figure 4:**
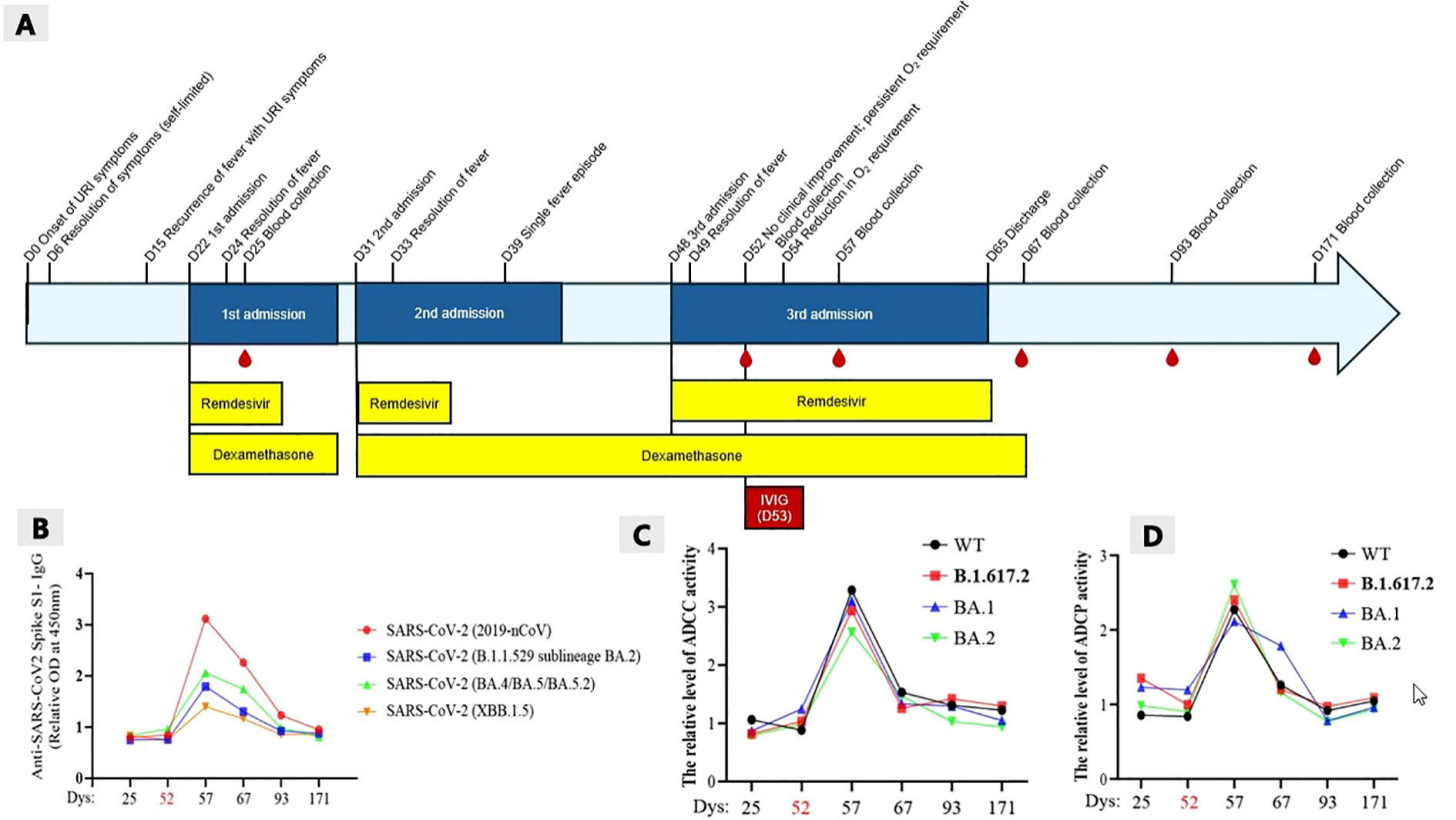
Clinical timeline and SARS-CoV-2–specific immune responses. **(A)** Timeline of the patient’s clinical course, including hospitalization periods, administration of therapeutic agents (remdesivir, dexamethasone, and IVIG), and blood collection time points (indicated by blood drop icons). **(B)** Longitudinal IgG titers against SARS-CoV-2 spike S1 protein from the wild-type strain and Omicron sublineages before and after IVIG. **(C, D)** Kinetics of Fc-mediated effector functions, including antibody-dependent cellular cytotoxicity (ADCC) **(C)** and antibody-dependent cellular phagocytosis (ADCP) **(D)** against the indicated SARS-CoV-2 variants. Red labels on the x-axis denote the baseline measurement immediately before IVIG administration (day 53).

During the 15-day interval between admissions, the patient experienced a persistent cough and intermittent low-grade fever. On Day 48, her condition acutely worsened with high fever and severe dyspnea, requiring 3 L/min of supplemental oxygen. Laboratory tests showed a WBC count of 7,310/mm^3^ (lymphocytes 4.5% absolute lymphocyte count 329/mm^3^) and markedly elevated inflammatory markers, notably ferritin (2,422 ng/mL) and LDH (620 U/L). The persistence of the infection was again molecularly confirmed; RT-PCR remained positive with Ct values of 28.26 (E), 29.26 (RdRP/S), and 27.78 (N) (Day 48). Chest CT and follow-up radiography showed progressive multifocal consolidations and GGOs (**Figures 3A–D**). Given the failure of repeated standard antiviral courses, salvage IVIG (2 g/kg) was administered on Day 53. Following treatment, the patient showed rapid clinical stabilization and fever resolution. A chest radiograph at discharge confirmed significant resolution of the lung lesions (**Figure 3E**), and she remained stable throughout a 6-month follow-up period (**Figure 4A**).

### 2.3 Dynamic changes in IgG antibody titer, ADCC, and ADCP

Longitudinal assessment showed consistent seronegativity until Day 52 (one day prior to IVIG), with anti-nucleocapsid (N) IgG at 0.32 IU/mL (cutoff >1.0) and anti-spike (S1) IgG at 1.5 IU/mL (cutoff >10). During this period, specific IgG against Omicron variants (wild-type, BA.2, BA.4/5, and XBB) and Fc-mediated functions (ADCC/ADCP) were also negligible. Following IVIG administration on Day 53, both antibody titers and effector functions increased markedly, peaking at one week post-infusion before gradually declining (**Figures 4B–D**).

## 3 Discussion

This case demonstrates the successful use of IVIG in treating recurrent COVID-19 pneumonia in a patient with rituximab-induced B-cell depletion. Despite standard therapy, the patient experienced repeated episodes of respiratory deterioration and radiographic pneumonia that improved only after IVIG administration. Notably, the clinical recovery was closely synchronized with a robust increase in SARS-CoV-2–specific IgG titers and the restoration of Fc-mediated effector functions, including ADCC and ADCP. These findings provide a plausible mechanistic hypothesis for IVIG efficacy in this vulnerable patient population.

As recently highlighted by Carvajal et al. (14), patients on anti-CD20 therapy face a significantly increased risk for prolonged viral shedding and disease relapse (5–7, 15, 16). In our case, the patient’s inability to generate an endogenous response was circumvented through passive antibody transfer. While convalescent plasma (CP) was initially considered as a therapeutic option—particularly given a potential donor identified with robust antibody titers (anti-S1 IgG >16,000 IU/mL)—its application was precluded by logistical complexities and the time required for specialized processing. Given the patient’s rapidly deteriorating clinical state, IVIG was prioritized as an immediate, standardized ‘off-the-shelf’ intervention. Unlike CP, IVIG provided consistent, high-titer pooled antibodies from a broad donor base, leading to rapid clinical recovery that coincided with the restoration of circulating SARS-CoV-2–specific IgG (3, 4, 17, 18).

Beyond conventional neutralization, IVIG is known to exert pleiotropic immunomodulatory effects, including the attenuation of hyperinflammation, regulation of T-cell responses, and blockade of Fc receptors (11, 13). While neutralization primarily prevents the virus from entering new host cells, Fc-mediated functions are essential for the elimination of cells that are already infected (3, 19–21). Specifically, ADCC enables natural killer cells to recognize and destroy virus-infected cells, effectively shutting down the ‘factories’ of viral replication. Meanwhile, ADCP promotes the phagocytic clearance of opsonized viral particles and infected cellular debris by monocytes and macrophages, which is crucial for resolving tissue inflammation (19–21). In this context, we hypothesize that the clinical recovery observed in our patient resulted from a synergistic interplay between these broad immunomodulatory actions and a targeted Fc-mediated antiviral response.

While the observed increase in ADCC and ADCP could partially reflect a non-specific effect of pooled immunoglobulins, several lines of evidence support the potential significance of a targeted response in this case. Unlike global immunoglobulin elevation, our functional assays specifically demonstrated a robust increase in SARS-CoV-2 Spike-specific ADCC and ADCP activities. As highlighted by Moradimajd et al., IVIG’s therapeutic potential in viral infections often involves the restoration of Fc gamma receptor-mediated clearance (13). This hypothesis is further strengthened by Zimmerman et al. (10), who demonstrated that the *in vivo* efficacy of IVIG is primarily dependent on these functional antibody pathways. Furthermore, the marked clinical improvement and the resolution of persistent COVID-19 pneumonia showed a clear temporal association with the recovery of these antigen specific functional titers, which aligns with previous reports of successful IVIG therapy in B-cell-depleted hosts (13, 15, 16).

The discrepancy between our findings and several reports that showed no benefit of IVIG in COVID-19 can be explained by two key factors (22–25). First antibody potency in IVIG batches has increased progressively as population immunity evolved. While we did not perform lineage-specific genomic sequencing for this patient, the infection occurred in July 2023, a period when the Omicron XBB lineage (notably EG.5) was the predominant circulating variant in South Korea (26). The IVIG lot used was released on July 18, 2023, implying it likely contained antibodies specific to the then-circulating strains (27). Second, there is a fundamental difference in expected efficacy between immunocompetent individuals and patients with B-cell depletion. In immunocompetent patients, IVIG may be redundant as they already produce endogenous antibodies (3, 20). This explains why large-scale trials in unselected populations often failed to show consistent benefits (28, 29). Conversely, for B-cell depleted patients with an absolute antibody deficit, IVIG serves as a critical ‘functional bridge,’ providing the essential humoral components required for viral clearance (13, 15, 16).

However, these findings must be interpreted in light of certain limitations. First, as a single-patient case report, our findings preclude definitive causal inference and require validation in larger cohorts to confirm the efficacy of IVIG in patients with B-cell depletion. Second, we did not perform longitudinal virologic assays, such as serial Ct value tracking following IVIG administration. The absence of these data limits the definitive assessment of virological clearance and makes it challenging to strictly distinguish between a direct treatment response and the natural resolution of a persistent or relapsing clinical course. Third, quantitative B-cell enumeration was not serially performed, which precludes a precise confirmation of the extent of B-cell depletion and the timing of B-cell recovery. However, the patient’s consistent seronegativity for both anti-N (0.32 IU/mL) and anti-S1 (1.5 IU/mL) antibodies, even after nearly 52 days of infection, provides compelling functional evidence of profound rituximab-induced humoral deficiency, essentially serving as a surrogate for B-cell depletion.

Acknowledging these constraints, several key strengths of this study warrant mention. The longitudinal assessment of both antibody titers and Fc mediated effector functions enabled a precise temporal correlation between immunologic restoration and clinical improvement. Integrating detailed clinical data with comprehensive functional assays provides a potential insights into the immunological processes that have been limited in previous case reports (13, 15, 16). Overall, this case suggests that exogenous antibody supplementation could be considered as a potential therapeutic adjunct for persistent COVID-19 in patients with impaired endogenous immune responses.

In conclusion, this case highlights IVIG as a potential adjunctive therapy for persistent COVID-19 in B-cell-depleted patients. The temporal association between restored Fc-mediated effector functions and clinical recovery underscores the critical role of functional antibodies in viral clearance. These observations provide mechanistic support for passive immunotherapy and warrant further prospective studies to optimize dosing and efficacy in this vulnerable population.

## 4 Methods

Supplementary Material provides the full methodological details of the functional assays. Serologic analysis was conducted on longitudinally collected serum samples. SARS-CoV-2–specific IgG titers against the wild-type strain and Omicron variants (BA.2, BA.4/5, XBB.1.5) were measured using enzyme-linked immunosorbent assay (ELISA). Functional Fc-mediated immunity was assessed using luciferase-based reporter assays to quantify ADCC (Jurkat-Lucia™ NFAT–CD16) and ADCP (Jurkat-Lucia™ NFAT–CD32) activity against spike-expressing target cells.

## Data Availability

The raw data supporting the conclusions of this article will be made available by the authors, without undue reservation.
