# Supplementary material for: Case Report: Successful treatment of recurrent COVID-19 with intravenous immunoglobulin in a patient with rituximab-induced B-cell depletion and restoration of Fc-mediated effector functions
Source: Front Immunol. 2026 May 25;17:1797070. doi: 10.3389/fimmu.2026.1797070 (PMC13243378; doi:10.3389/fimmu.2026.1797070)
Supplement: Supplementary file 1 [file Table1.docx]

Supplementary Material

# Cell Culture, Chemicals, and Proteins

We purchased 293T (CRL-3216), THP-1 (TIB-202), and A549 (CRM-CCL-185) cells from ATCC (Manassas, VA, USA). Jurkat-Lucia^TM^ NFAT–CD32 and Jurkat-Lucia^TM^ NFAT–CD16 reporter cells were purchased from InvivoGen (CA, USA). Dulbecco’s modified Eagle’s medium (DMEM), RPMI-1640, phosphate-buffered saline (PBS), and penicillin–streptomycin (Pen/Strep) were obtained from WELGENE (Daegu, Korea). Iscove’s modified Dulbecco’s medium (IMDM) was purchased from InvivoGen (CA, USA). We maintained 293T cells and A549 cells in DMEM supplemented with 10% fetal bovine serum (FBS) and 1% Pen/Strep at 37℃ in a 5% CO_2_ incubator. THP-1 cells were cultured in RPMI-1640 containing 0.05-mM β-mercaptoethanol (Sigma-Aldrich, MO, USA) and 10% FBS under the same conditions. Jurkat-Lucia^TM^ NFAT–CD32 and Jurkat-Lucia^TM^ NFAT–CD16 cells were maintained in IMDM supplemented with 10% FBS, 100 μg/mL Normocin^TM^, and 1% Pen/Strep at 37℃ with 5% CO_2_. Lipofectamine^TM^ 3000 transfection reagent was purchased from Thermo Fisher Scientific (Waltham, MA, USA). SARS-CoV-2 (2019-nCoV) Spike S1 (45951-V08H), SARS-CoV-2 (B.1.1.529, sublineage BA.2) Spike S1 (40591-V08H43), SARS-CoV-2 (BA.4/BA.5/BA.5.2) Spike S1 (40591-V08H46), and SARS-CoV-2 (XBB.1.5) Spike S1 (40591-V08H47) proteins were purchased from SinoBiological (Beijing, China).

# Generation of A549-ACE2 Stable Cells

pLENTI-hACE2-puro (#155295), pMD2.G (#12259), and psPAX2 (#12260) plasmids were purchased from Addgene (Cambridge, MA, USA). We transfected 293T cells with 4.3 μg pLENTI-hACE2-puro together with lentiviral helper plasmids (9 µg psPAX2 and 4 μg pMD2.G). After 72 h, viral supernatants were collected, filtered, and used to transduce confluent A549 cells. Transduced cells were selected with puromycin (3 μg/mL) for 48 h to generate a stable A549-ACE2 cell line.

# SARS-CoV-2 S1 Spike IgG ELISA

SARS-CoV-2 S1 proteins (wild-type, BA.2, BA.4/BA.5/BA.5.2, XBB.1.5) were coated onto 96-well immunoplates (Thermo Fisher Scientific, Waltham, MA, USA) at 2 μg/mL and incubated overnight at 4℃. Plates were then blocked with 1× PBS containing 1% bovine serum albumin for 1 h at room temperature. After three washes with 1× PBST, diluted plasma samples (1:20,000) were added and incubated for 2 h at room temperature. Plates were washed five times with 1× PBST and incubated with horseradish peroxidase–conjugated anti-human IgG (1:20,000, Jackson ImmunoResearch, West Grove, PA, USA) for 1 h at room temperature. After seven additional washes, plates were developed with 3,3′,5,5′-tetramethylbenzidine substrate (Sigma-Aldrich, MO, USA) for 30 min, and the reaction was stopped using a stop solution (Sigma-Aldrich). Optical density (OD) values were measured at 450 nm using a SPARK multimode reader (TECAN, Switzerland). Cutoff values were determined by analyzing OD values from 10 SARS-CoV-2–negative control samples. The cutoff for each assay was defined as the mean OD plus three standard deviations.

**4 Antibody-Dependent Cellular Phagocytosis Assay**

Plasma samples were heat-inactivated at 56°C for 30 min and diluted 1:100 in medium. A549–SARS-CoV-2 spike cells (5.0 × 10^5^ cells/mL) were seeded onto 96-well plates and incubated for 24 h at 37°C. After three washes with 1× PBST, diluted plasma was added and incubated for 24 h at 37°C. Jurkat-Lucia^TM^ NFAT–CD32 cells (1.0 × 10^6^ cells/mL) were then added and incubated for 6 h at 37°C. Luciferase activity was quantified using QUANTI-Luc^TM^ (InvivoGen, CA, USA), and luminescence was measured with a SPARK multimode reader (TECAN, Switzerland).

# Antibody-Dependent Cellular Cytotoxicity Assay

Plasma samples were heat-inactivated at 56°C for 30 min and diluted 1:100 in medium. A549–SARS-CoV-2 spike cells (5.0 × 10^5^ cells/mL) were seeded onto 96-well plates and incubated for 24 h at 37°C. After three washes with 1× PBST, diluted plasma was added and incubated for 24 h at 37°C. Jurkat-Lucia^TM^ NFAT–CD16 cells (1.0 × 10^6^ cells/mL) were then added and incubated for 6 h at 37°C. Luciferase activity was quantified using QUANTI-Luc^TM^ (InvivoGen, CA, USA), and luminescence was measured with a SPARK multimode reader (TECAN, Switzerland).

# Statistics

Statistical analyses were conducted using Student’s *t*-test in GraphPad Prism 10.1.2 (San Diego, CA, USA). Statistical significance was defined as **p* < 0.05, ***p* < 0.01, and ****p* < 0.001.
